# Bile Duct Strictures Caused by Solid Masses: MR in Differential Diagnosis and as a Prognostic Tool to Plan the Endoscopic Treatment

**DOI:** 10.1155/2013/729279

**Published:** 2013-11-05

**Authors:** Tomasz Gorycki, Michał Studniarek

**Affiliations:** Department of Radiology, Medical University of Gdańsk, 7 Dębinki Street, 80-950 Gdańsk, Poland

## Abstract

The aim of the study was to assess how realiable is differential diagnosis and prognosis for endoscopic treatment with MR signal characteristics as the qualitative parameter and magnetic resonance cholangiopancreatography (MRCP) images in cases of bile duct obstructions caused by solid masses. *Material and Methods*. Retrospective study of MR and MRCP images in 80 patients (mean age 58 ys) was conducted. Mean signal intensity ratio (SIR) from planar MR images and MRCP linear measurements were compared between benign and malignant lesions and in groups including the size and number of stents placed during ERCP (< 10 F <) in 51 cases in which ERCP was performed. *Results*. Significantly higher SIR values were encountered in malignant lesions in T2W images (*r* = 0,0003) and STIR T2W images (*r* = 0,0002). Malignant lesions were characterised by longer strictures (*r* = 0,0071) and greater proximal biliary duct dilatation (*r* = 0,0024). High significance for predicting ERCP conditions was found with mean SIR in STIR T2W images and stricture length. *Conclusion*. Probability of malignancy of solid lesions obstructing biliary duct increased with higher SIR in T2W images and with longer strictures. Passing the stricture during ERCP treatment was easier and more probable in cases of shorter strictures caused by lesions with higher SIR in STIR T2W images.

## 1. Introduction

There is an increase of number of patients undergoing magnetic resonance (MR) prior to endoscopic retrograde cholangiopancreatography (ERCP) [1, 2]. MR with magnetic resonance cholangiopancreatography (MRCP) offers wide range of qualitative and quantitative parameters characterising solid lesions resulting in biliary strictures allowing for better planning of intervention methods to reduce number of complications [1, 2].

Magnetic resonance imaging (MRI) plays an important role in evaluation of hepatobiliary system in all hepatic, suprapancreatic, and pancreatic segments. In many comparative imaging studies MRI shows similar or even higher diagnostic accuracy in focal hepatic lesions detection than multidetector computed tomography (MDCT, CT) [3–5]. When surroundings of the biliary tree in hepatic segment are concerned, MRI appears to have higher ability of precise characterization of observed hepatic lesions [3–5]. Also in the imaging of the pancreatic region of biliary duct MRI is significantly superior to CT in both detecting and excluding malignant conditions [4]. Conventional T1-weighted and T2-weighted images allow the evaluation of extraductal soft tissues with increasing diagnostic accuracy by demonstrating the extension of masses surrounding biliary duct [6–8].

Usually the value of differential diagnosis based on T1- and T2-weighted images and Gd-DTPA enhanced T1-weighted images, and MR cholangiographic images were determined for each of these techniques alone. Until now many authors have analysed the technique of examination, diagnostic features of benign versus malignant lesions in parenchymal regions around biliary ducts, focusing on qualitative analysis of signal intensity [4–8]. Others used semiquantitative scales in order to establish certain features of MR sequences including liver signal-to-noise ratio, contrast-to-noise ratio or lesionliver contrast-to-noise ratio [9]. Kim et al. have undertaken a trial to determine the value of conventional T1- and T2-weighted images and Gd-DTPA enhanced MR images as a supplement to MR cholangiographic images in differentiation of benign from malignant lesions that cause biliary dilatation asking the observers to review the images using a point scale and assign a confidence level to their evaluation of the cause of biliary abnormality [6]. Although MRCP provides the same imaging information as direct cholangiography, used alone it has limited specificity for the diagnosis of malignant strictures [10]. In everyday practice a combination of MRCP and cross-sectional MRI should be performed in assessment of pancreatobiliary disorders [1, 6, 7, 10–12]. 

The purpose of this study was to assess how realiable is the differential diagnosis and prognosis for endoscopic treatment on the basis of signal characteristics from plane MR images and MRCP images in cases of bile duct obstructions caused by solid masses.

## 2. Materials and Methods

T1W (SE 500/10), T2W (UTSE/RC 2500/100), STIR T2W (TSE 2500/100), and T1W (SE 500/10) contrast-enhanced (Gd-DTPA) nonbreathhold, respiratory gated MR images including the entire hepato-pancreatic region as well as cholangiopancreatographic MR images (3DTSE 1800/700) were acquired in axial and coronal planes in 80 patients (mean age 58,3) directed to MR examination after ultrasonography disclosing cholestasis. In 51 patients endoscopic retrograde cholangiopancreatography (ERCP) was performed with Pentax ED-3440T, and stents to provide the optimal conditions of biliary drainage have been placed. The aid was to insert two or one at least of 10 F size. However, in 14 cases of tight biliary strictures only single 7 F stents were successfully inserted. All ERCP procedures were performed by experienced interventional gastroenterologists. 

In 53 cases malignant lesions have been confirmed in surgery and biopsy to be a reason of biliary obstruction. There were as follows: 25 cases of pancreatic cancer, 11 cases of hepatic metastases, 9 cases of cholangiocarcinoma, 3 cases of metastatic nodules in the hilar region, 3 cases of carcinoma of the gall bladder, and 2 cases of hepatocellular carcinoma. Among 27 cases of benign conditions of inflammatory origin, 21 patients underwent follow-up MRI examinations for a 2 year period, and therapeutic procedures both endoscopic or during open surgery were performed in cases of biliary ducts dilatation or when anastomosis was required. 

In 6 patients the benign origin has been proved in 2-year period of clinical observation. 

Mean signal intensity ratio (SIR) of benign versus malignant lesions when countable area of mass was detected around bile duct strictures as well as the linear measurements covering stricture morphology from MRCP images has been compared. Also a correlation with size of stents inserted during endoscopy was analysed.

**Figure 1:**
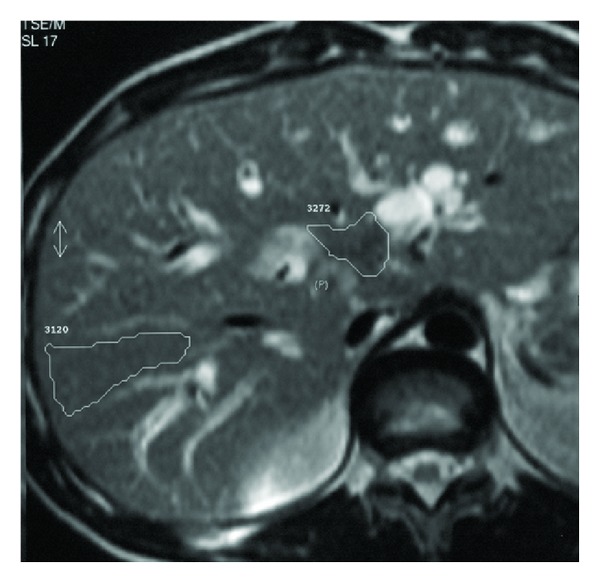
Axial plane scan for ROIs drawings. A case of hilar cholangio cell carcinoma.

**Figure 2:**
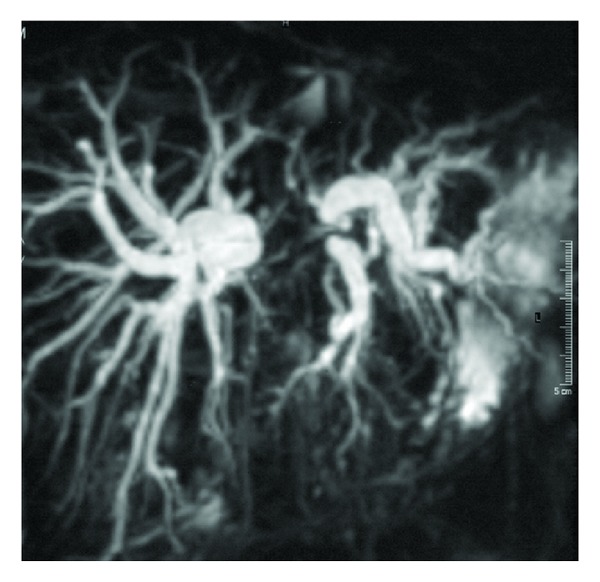
Corresponding MRCP image describing stricture morphology.

Images subjected to analysis represented strictly the same plane with well-defined lesion and corresponded to the level of biliary obstruction observed on MRCP images. Quantitative analysis was performed with both operator-defined region of interest (ROI) measurements of mean signal intensity of lesion and its background and linear measurements from MRCP images concerning morphology of the biliary stricture a.i. stricture length, stricture width, proximal ductal dilatation, and distance from stricture to hepatic ducts junction. For the liver and pancreatic lesions ROIs for measuring SI of background were put in areas devoid cystic structures, large vessels, and inhomogeneities in organs of their origin accordingly (Figures 1 and 2). For lesions causing strictures of hilar and suprapancreatic segments of biliary ducts ROIs for background SI were taken like for hepatic lesions. Signal intensity ratio (SIR) calculated from these measurements as well as MRCP linear measurements were compared in two groups including benign and malignant lesions as well as in two groups with the inclusion of the size of stents (one stent size less than 10 F versus 10 F or more than one stent placed) in 51 cases in which stents have been inserted. Statistical analysis of the obtained data was performed with the use of Kruskal Wallis test. The usefulness of the tests for purposes of differential diagnosis, accuracy, and predictive values was established based on analysis of receiver operating curves (ROC) counted for every feature.

## 3. Results 

Significantly higher mean SIR was encountered in malignant lesions in T2W (*r* = 0, 0003) and STIR T2W images (*r* = 0, 0002). Malignant lesions were also characterised by longer strictures (*r* = 0, 0071) and larger diameter of biliary ducts proximatly to obstruction (*r* = 0, 0024), whereas SIR counted from T1W images with and without contrast enhancement (*P* = 0,4395, *P* = 0,6163) and stricture thickness (*P* = 0,4266) did not differ significantly between benign and malignant lesions. 

**Figure 3:**
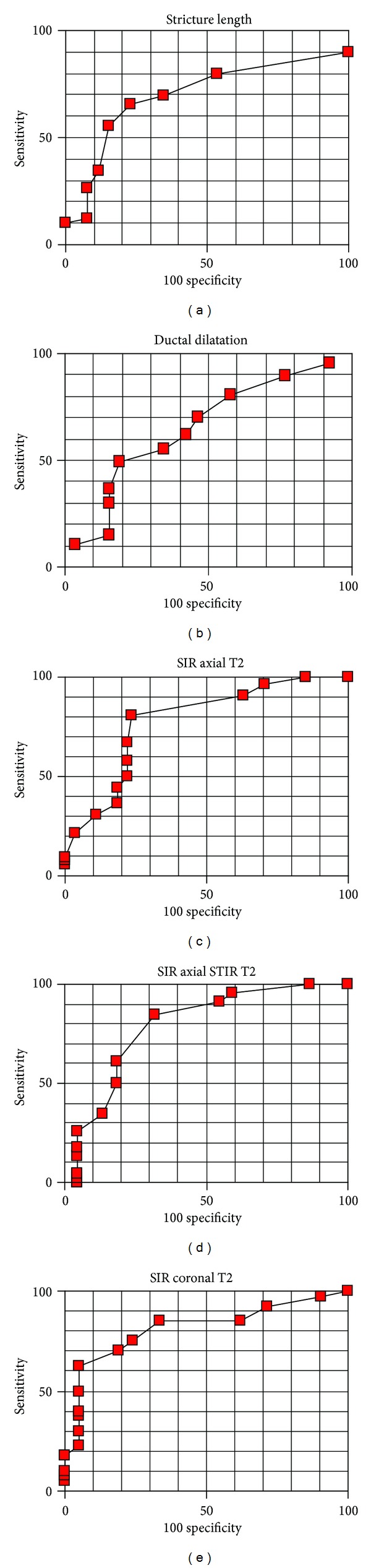
ROC curves for differential diagnosis between benign and malignant lesions with MRCP stricture morphology and SIR.

**Figure 4:**
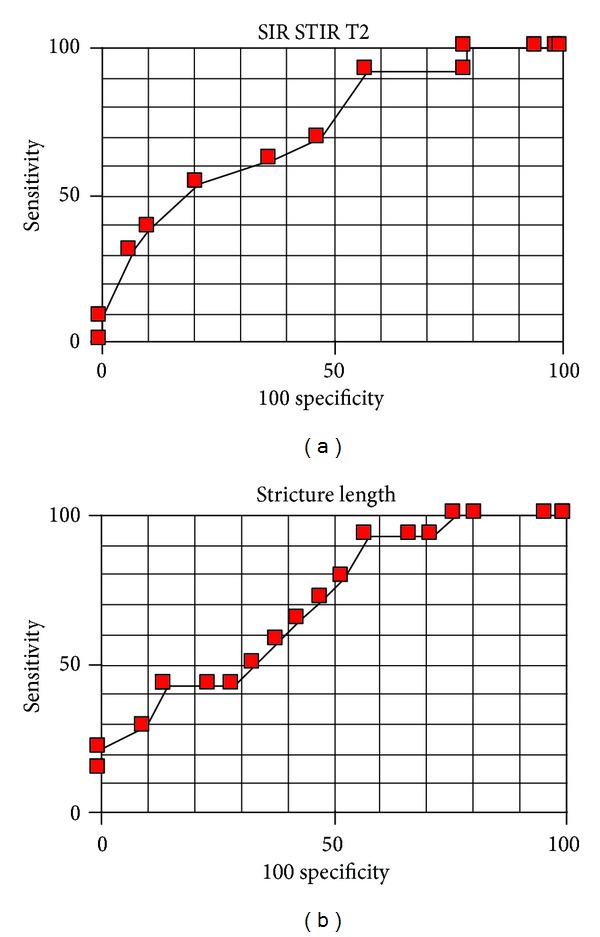
ROC curves for prognosis of endoscopic treatment based on SIR from STIR T2W images and stricture length.

When the size and number of stents were taken into account significantly distinguishing features were—mean SIR in STIR T2W (*P* = 0,0224) images and stricture length in MRCP images (*P* = 0,0312). Therefore it was easier to pass a stricture during ERCP when the lesion leading to the stricture showed higher SIR in STIR T2W images and in cases of shorter strictures. SIR counted from T1W images (*P* = 0,7121), T1W images with contrast enhancement (*P* = 0,4502), and T2W images without fat saturation (*P* = 0,7121) did not differ significantly between groups of lesions around strictures treated with ERCP procedure. These relations were also described by ROC curves—Figures 3 and 4. Account of results obtained including predictive values is presented in Tables 1, 2, and 3.

## 4. Discussion

For the lesions associated with obturation of the bile duct reference standards used to prove the etiology and for comparison were surgery, a biopsy confirming malignancy, or the clinical course during followup (at least 12 months) in cases without histopathologic proof of malignancy [13–15]. In this study in 53 cases malignant lesions have been confirmed in surgery and biopsy to be a reason of biliary obstruction. Among 24 cases of benign conditions, mostly of inflammatory origin, 21 patients underwent follow-up MRI examinations for a 2-year period, and therapeutic procedures both endoscopic or during open surgery were performed in cases of biliary ducts dilatation or when anastomosis was required. In 6 patients the course of the disease proved the benign origin in 2-year period of clinical observation. 

The sensitivity, specificity, and accuracy of MRCP for detecting and locating bile duct strictures were 85–100%, 71–100%, and 70–100% among the studies [12–18]. However, MRCP alone had a limited ability to reveal concurrent intraductal cholangiocarcinoma associated with hepatolithiasis. Also nondilated poorly visualised bile ducts distally from strictures can lead to misdiagnosis [13, 16–18]. In 3D MRCP the use of water as a natural contrast in duodenum and evaluation of both (maximum intensity projection) MIP and source cholangiopancreatographic images are the simplest methods to decrease the number of misdiagnoses in these cases [16, 18]. The influence of bile duct dilatation and presence of gas and fluid in the duodenum on sensitivity in the diagnosis of localization and cause of biliary duct pathologies are also common in ultrasonography performed in most cases prior to magnetic resonance [19, 20].

Analysis of imaging variables at MR images with characteristics at MRCP images together was found to be the best predictor of malignancy of the bile duct stricture [6, 7, 18]. MR with MRCP proved the same diagnostic value as ERCP for visualizing the bile ducts, replacing ERCP as the primary investigation in patients with multiple risk factors; this would reduce the numbers of patients exposed to the risks of ERCP [14, 15, 21]. 

In this study SIR was counted on the basic MR images (T1W, T2W, STIR T2W) which proved similar diagnostic value between different MR systems whereas modified T2W images could represent different diagnostic value strongly dependent on the MR system and kind of sequence used in the study [6–8]. 

Among different factors used to describe signal characteristics of lesions in liver and pancreas the most accurate for the purposes of differential diagnostics is signal intensity ratio based on mean signal intensity (SIR) measurements performed in operator's dependent regions of interest [6, 8, 9]. Minimum pixels number covered with ROI enough to avoid stronger influence of the partial volume effect differs between studies from 25 to 100 [2, 11]. In this study mean SI of the smallest lesion was counted from ROI consisting of 92 pixels (197 mm^2^). In the study no significant differences in SIR values between malignant and benign lesions counted from T1-weighted and Gd-enhanced T1-weighted images were found. The use of fast spin echo sequences allowed the increase of TR which has strong influence on T2W contrast and with only slight prolongation of time of acquisition. These sequences allow better tissue separation within T2 contrast area [6, 7, 9]. In fast spin echo sequences transfer of magnetization has strong influence on solid tissue areas and minimal influence on fluid-reach areas like poorly drained or edematous areas and with blood plasma leakage from vessels gathered around lesion causing them to present as areas of relatively high signal intensity [22–24]. This phenomenon is also easily observed, for example, in renal parenchyma built with tubular fluid-filled structures. In this way, it is possible mechanism for relative signal intensity increase measured with SIR in areas of malignant infiltration near tubular structures like biliary or pancreatic ducts. It was observed in all T2-weighted images as a difference between groups of malignant versus benign conditions in this study (*P*:  0,0002-0,0003). Also the relative signal intensity of malignant metastatic lesions in the area of hepatoduodenal ligament was higher than that of surrounding tissues in T2-weighted images because of the long T2 especially in sequences with fat saturation [22]. Assessment of ROC curves concerning T2-weighted sequences (with or without fat saturation and in both planes) shows that the cut-off levels are very similar (1,1006–1,1173) with similar accuracy counted for their values (75,4–79,7%). Therefore, no significant differences in information supplied by presented T2-weighted images have been stated.

Also submitted to analysis were MRCP MIP and source images. Besides the fact that MRCP requires neither contrast medium administration nor biliary and pancreatic intervention, this technique presents further important advantages. The biliary tree is shown without dilatation effect due to pressure and choleretic effect of contrast medium used for ERCP or percutaneous cholangiography, and it is also possible to obtain images of the whole biliary tree even in cases of critically tight strictures or complete obstruction that would prevent passing them with leaders during endoscopy or percutaneous cholangiography as carrying a high risk of perforation [2, 14, 15, 17, 22]. MRCP not being the therapeutic procedure itself allows to plan the optimal way of drainage. The message of these relativities follows the general tendency to decrease the number of diagnostic ERCP and to perform ERCP as therapeutic procedure only [2, 14, 15, 17, 22]. 

 It is worth mentioning that presumably there are two factors changing the accuracy rate of MRCP images in determining the nature of biliary obstruction: the biliary duct dilatation and the use of cross-sectional scans for analysis [16–18, 23], what corresponds to the results obtained by Pamos et al. [24]. In his study higher sensitivity and specificity in the diagnosis of malignancy were obtained with MRCP in cases of dilated biliary ducts. As shown in this study SIR counted from T2W images could increase to some extent the accuracy of diagnosis of malignant lesion causing the biliary obstruction, acting as additional factor to be assessed together with MRCP images. 

STIR T2-weighted images had the highest predictive value when assessing the conditions for endoscopic treatment, when the size and number of stents were taken into account. Masses causing biliary strictures characterised by higher SI in STIR T2-weighted images proved to be easier to pass with stents during endoscopic procedures. Tight strictures associated with the presence of dense fibrous tissues show as low intensity areas in T2-weighted images especially when fat saturation sequence was applied. From morphologic measurements analysed in MRCP images only the stricture length was associated with the size of stents with which the stricture was passed. As one could expect longer strictures caused more difficulties during endoscopy. However the stronger feature as shown on ROC curves for the purposes of the evaluation of stenting conditions seems to be SIR measured in STIR T2-weighted images as shown in Table 3. 

Three limitations of this study must be considered. First, no diffusion weighted images (DWI) were performed in the group taken to analysis, so in order to establish the relationship between signal profile in DWI and conditions during ERCP the study should be continued. Second, there were analysed less cases of benign lesions (27/80) versus malignant (53/80). Presumably, it was caused by the fact that some of the cases were diagnosed with CT following ultrasound without MRI. The third limitation of the work was that no 2D MRCP images were used for analysis; however, they are used in every day practice to the same extent of those of 3D MRCP, but in many cases better understanding of the complex biliary strictures is possible due to analysis of both source images and the reconstructions, and this is possible only with 3D MRCP technique.

## 5. Conclusions

Probability of malignancy of solid lesions obstructing biliary duct increased with higher SIR in T2W images and with longer strictures observed in MRCP images. Introducing a larger size stents was more probable in cases of lesions showing higher SIR in STIR T2W images and in case of shorter strictures. These features could be additional parameters taken into consideration when conditions for endoscopic treatment are being discussed in order to reduce complications during ERCP.

## Figures and Tables

**Table 1:** PPV and NPV in detecting the malignancy with SIR calculated for cut-off levels.

Image	ROC level	Accuracy %	PPV %	NPV %
Axial T2W	1,1006	79,7	87,5	67,7
Axial STIR T2W	1,1010	79,4	84,8	65,2
Coronal T2W	1,1173	75,4	85,7	61,5

**Table 2:** PPV and NPV in detecting the malignancy with MRCP morphology calculated for cut-off levels.

	ROC level	Accuracy %	PPV %	NPV %
Stricture length (mm)	21,3	69,3	84,2	54,1
Ductal dilatation (mm)	12,3	58,9	74,3	60,7

**Table 3:** PPV and NPV when stricture length and SIR STIR T2 were used to forecast the conditions for biliary stenting.

	ROC level	Accuracy %	PPV %	NPV %
Stricture length (mm)	22,1	60	50	70,6
SIR STIR T2	1,244	68,8	63,6	71,4
